# The Effect of Inclines on Joint Angles in Stroke Survivors During Treadmill Walking

**DOI:** 10.3389/fneur.2022.850682

**Published:** 2022-04-11

**Authors:** Xin Zhang, Yanting Lu, Jung Hung Chien, Chenlei Fu, Zhe Zhou, Hua Li, Gongwei Hu, Tianbao Sun

**Affiliations:** ^1^Department of Rehabilitation, School of Medicine, Tongji University, Shanghai, China; ^2^Shanghai Rehabilitation Center, Shanghai First Rehabilitation Hospital, Shanghai, China; ^3^JC Movement Science LLC, Omaha, NE, United States; ^4^Nuerological Intensive Rehabilitation Department, Shanghai First Rehabilitation Hospital, Shanghai, China

**Keywords:** joint angles, gait, stroke, paretic leg, incline

## Abstract

Stroke severely affects the quality of life, specifically in walking independently. Thus, it is crucial to understand the impaired gait pattern. This gait pattern has been widely investigated when walking on a level treadmill. However, knowledge about the gait pattern when walking on inclines is scarce. Therefore, this study attempted to fulfill this knowledge gap. In this study, 15 stroke survivors and 15 age/height/weight healthy controls were recruited. The participants were instructed to walk on three different inclines: 0°, 3°, and 6°. The participants were required to walk on each incline for 2 min and needed to complete each incline two times. The dependent variables were the peak values for ankle/knee/hip joint angles and the respective variability of these peak values. The results showed that an increment of the incline significantly increased the peak of the hip flexion and the peak of the knee flexion but did not affect the peak values of the ankle joints in the paretic leg in these stroke survivors. In comparison with the healthy controls, lower hip extension, lower hip flexion, lower knee flexion, and lower ankle plantar flexion were observed in stroke survivors. A clinical application of this work might assist the physical therapists in building an effective treadmill training protocol.

## Introduction

Stroke is the third leading cause of death in China (1). The death rate due to stroke was 149.49 per 100,000, resulting in 1.57 million deaths in 2018 in China (1). However, only 10% of the stroke survivors could recover completely based on the report from the American Stroke Association (2). Therefore, the quality of life was severely affected in these stroke survivors, specifically in walking independently, due to the reduced peak of the knee flexion angle (3, 4) and the reduced peak of the ankle dorsiflexion angle (3) in the paretic leg.

Treadmill training has been widely used to improve the recovery rate in these stroke survivors, and the results indeed find that treadmill training increases gait speed and walking endurance (5–10). However, a review with 44 relevant studies, involving 2,658 patients with stroke, concludes that the stroke survivors who receive gait training are not likely to improve their ability to walk independently compared to those who do not receive this treadmill training, except their gait speed and gait endurance (11). This review further suggests that increasing training intensities might be required, such as walking on the inclines (11). To verify this suggestion, a study trains the patients with stroke to walk on a 10% inclined treadmill in a 20-min session, and 12 sessions within a month are provided (12). The results of the previous study show increments (improvements) in the walking velocity, the step length in the paretic leg and non-paretic leg, and the hip range of motion.

The gait pattern in the stroke survivors while walking on the surface with different inclines has been investigated in a couple of studies. Phan et al. (13) observed that [1] the patients with stroke walked much slower than healthy controls, whether walking through a level or uphill surface (4.1°); [2] when healthy controls walked uphill, an increase of step length and a decrease of their cadence were observed compared to walking on a level surface; and [3] difficulties adjusting to their spatial-temporal gait parameters to adapt to different environments were found in the stroke survivors. Similarly, Moreno et al. (14) observed that there were no significant differences in the stride length and cadence when these stroke survivors were instructed to walk on the treadmill with different inclines (0°, 2.86°, and 5.74°). Even though there were no significant differences in the spatial-temporal gait parameters, the significant increases in the hip, knee, and ankle angles at the heel strike were observed to increase the inclines of the treadmill. However, the potential limitations in these above-mentioned studies were that only five homogeneous cycles were selected for the analysis and the selection of the lesion interval was wide (12, 14). Also, the effect of the asymmetric gait pattern was ignored, which is commonly found in the participants (48/54) (15).

In the past decades, a greater spatial-temporal gait variability has been associated with an unstable gait in patients with cerebellar ataxia (16) and in patients with cerebral white matter lesions (17). However, these studies majorly investigate the spatial-temporal gait variabilities, which only cover the variabilities in the transverse plane on the ground. However, the information of variability in the sagittal plane (joint angle variability) is scarce, specifically in stroke survivors. To the best of our knowledge, only a couple of studies have used the joint angle variability to identify multiple sclerosis (18), neuropathic patients (19), and the effect of a metronome on cerebellar stroke (20). The knowledge of how the joint angle variability changes in the patients with stroke during walking on different inclines is still unknown.

To answer the abovementioned knowledge gaps, there were three novelties of this study as follows: [1] to understand how different inclines affect the joint angles in the stroke survivors compared to healthy controls in paretic legs and non-paretic legs in 80 gait cycles; [2] the days since stroke was limited within 24 months, and [3] to understand the interaction of the joint angle variability between the effect of stroke and the effect of inclines. Based on the previous studies, a larger hip flexion/extension, larger knee extension, and larger ankle swing dorsiflexion would be observed in these stroke survivors in the paretic leg when walking on higher inclines. Additionally, the greater variability in the joint angles could be hypothesized in the stroke survivors compared to healthy controls, regardless of 0°, 3°, or 6° inclines.

## Materials and Methods

### Participants

A total of 15 stroke survivors and 15 healthy age-, height-, and weight-matched controls participated in this study (Table 1). All the participants were recruited from the Shanghai First Rehabilitation Hospital. The participants were screened during 24 months (January 2019 to November 2021). The stroke survivors with a single and unilateral hemiplegia (left side: 9, right side: 6), ability to walk a minimum of 10 m independently (21), a score of Functional Independence Measure Locomotor Item ≥ 5 (Phan et al., 2013), and the days from when the stroke was smaller than 24 months and larger than 6 months were included. The 15 stroke survivors did not wear any ankle–foot orthosis because they did not have any sign of spasticity around the ankle joint, which scored “0” based on the modified Ashworth Scale Score. These patients needed to tolerate a 30-min walking session under permission from their physical therapists. The exclusion criteria were orthopedic impairment, which caused the unstable gait, chronic pain in the musculoskeletal system affecting the gait, and the inability to perform the required tasks due to cognitive disorders. This study was approved by the Medical Ethics Committee of the Shanghai First Rehabilitation Hospital (YK-2020-01-020).

**Table 1:** Demographic characteristics of the individuals (*N* = 30).

**Characteristics**	**Stroke**	**Controls**
Age (years)	64.2 ± 12.68	60.4 ± 12.49
Gender	Male: 13, Female: 2	Male: 13, Female: 2
Body mass (kg)	69.43 ± 13.61	67.40 ± 12.09
Height (m)	1.70 ± 0.06	1.66 ± 0.07
Speed (m/s)	0.27 ± 0.14	0.67 ± 0.24^*^
Medication	Amlodipine besylate tablets, naoxueshu koufuye, clopidogrel sulfate tablets, clopidogrel sulfate tablets, metformin hydrochloride tablets	
Days since stroke (d)	221.60 ± 38.34	

*An independent t-test indicated no significant difference between the stroke survivors and healthy controls in weight, height, and age. However, an independent t-test showed that the healthy controls (0.27 m/s) walked much faster than the stroke survivors (0.67 m/s, p < 0.0001)*.

* *Significant difference between patients in stroke and controls*.

### Measures

**Figure 1:**
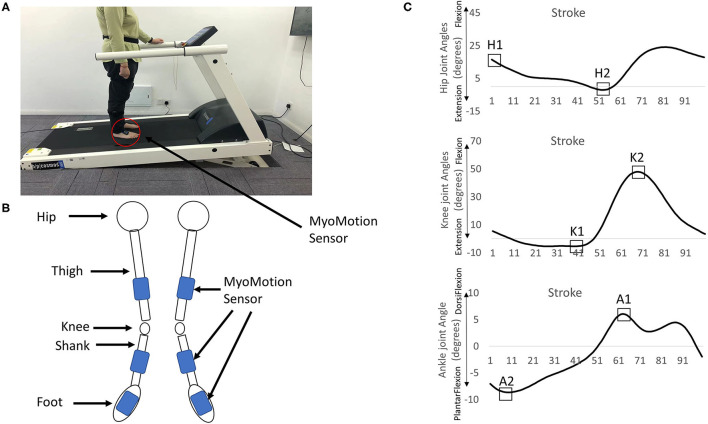
**(A)** The treadmill and the myoMotion sensors. **(B)** Six myoMotion sensors were attached on the thigh (frontal and distal half of the femur), the shank (front and slightly medial of the tibia), and the foot (upper foot, slightly below the ankle) through elasticated straps. **(C)** The focus of interests: [1] The peak of the hip flexion angle at the heel strike (H1), [2] the peak of the hip extension angle (H2), [3] the peak of the knee extension angle (K1), [4] the peak of the knee flexion (K2), [5] the peak of the dorsi-flexion angle (A1), and [6] the peak of the plantar flexion angle (A2) in the non-paretic/dominant leg and the paretic/non-dominant leg.

The joint angles (hip, knee, ankle) were obtained using Noraxon's myoMotion System (Noraxon Inc., Arizona, United States). This system contained two parts: a control unit and a set of 1–16 medical-grade inertial measure units (myoMotion Sensor). The myoMotion sensors were IMU-based (inertial measurement units). The control unit, which was connected to the computer, received the data collected from these myoMotion sensors through Bluetooth technology and the data were sent to the commercial software (myoRESEARCH 3.10.64, Noraxon, AZ, United States) for calculating the joint angles in a real-time fashion. Six myoMotion sensors were attached on the thigh (frontal and distal half of the femur), the shank (front and slightly medial of the tibia), and the foot (upper foot, slightly below the ankle) through elasticated straps (Figures 1A,B). Before providing walking trials to the participants, the participants were instructed to stand in an anatomical position consisting of standing upright, facing forward with the legs parallel to one another, putting arms at the sides, and none of the bones crossed. The joint angles at the anatomical position were recorded for 15 s and used as the offset base for other walking trials. This myoMotion system has been used to measure the joint angles during standing and gait (22–24) because the IMU-based system has the advantages of being low in cost, easy to set up, and portable (25). However, its validity and reliability might be questionable (24). More is discussed in the Limitation section. In this study, the participants were instructed to walk on the pressure-sensor-embedded treadmill (PhysTread Pressure Treadmill, Noraxon, AZ, United States). This treadmill contained a 150 x 50 cm running surface with 3,120 pressure sensors, and the recording rate was 100 Hz. By calculating the pressure distribution from these pressure sensors, the vertical force could be obtained. This vertical force was used to define the heel strike. The heel strike was defined when the vertical ground reaction force reached above a threshold level of 10 N and continuously exceeded this threshold for 40 ms but everywhere m/s (26). A gait cycle was defined from a heel strike to a following ipsilateral heel strike, and the time of each gait cycle was normalized to 0–100% of the gait cycle.

### Experimental Protocol

After the participants had consented, they were instructed to walk on the treadmill for familiarization. First, their preferred walking was defined as follows: [1] the participants stood on the side of the treadmill without stepping on the belt but withholding the handrail; [2] the treadmill was accelerated to 1.8 km/h; [3] the participants stepped on the belt holding the handrail. Once they felt confident, they were encouraged to walk without holding the handrail; [4] the participants were asked, “is this walking speed similar to your regular and comfortable walking speed?” If the participants felt too slow or too fast, a belt speed of 0.1 km/h was increased or decreased; [5] this preferred walking speed was checked repeatedly until reaching an agreement by the participants; [6] the participants then continued walking on this preferred walking speed for 2 min for familiarization. This similar procedure was followed by Chien et al. (26). Next, six walking trials lasting for 2 min were assigned to the participants in random combinations of the inclines (0°, 3°, and 6°, 18). The participants needed to walk each incline twice. It has been shown that the oxygenation level is higher in the areas of the prefrontal and sensorimotor cortex when walking on an uphill surface than if walking on a level surface (27). Thus, a 2-min mandatory rest (sitting) was provided between the trials to eliminate the fatigue (21) and to reset the sensation from the previous trials (28).

### Dependent Variables

The dependent variables in both the legs in 80 gait cycles, which was the lowest gait cycle made in 2 min in one stroke survivor, were as follows: [1] maximum hip flexion angle at heel strike (H1), [2] maximum hip extension angle (H2), [3] maximum knee extension angle (K1), [4] maximum knee flexion (K2), [5] maximum ankle dorsi-flexion angle (A1), and [6] maximum ankle plantar flexion angle (A2) in the non-paretic/dominant leg (NPL/DL) and the paretic/non-dominant leg (PL, NL, Figure 1C), and their respective variabilities. To identify the dominant leg, the healthy participants were asked, “If you want to kick a ball, which leg would you prefer?” All joint angles were time-normalized to 100% of the gait cycle each stride. The joint angle variability was calculated by the coefficient of variation (100 ^*^ standard deviation of each dependent variable/mean values of each dependent variable). A total of 80 gait cycles were suggested for calculating the variability (29).

### Statistical Analysis

An independent *t*-test was used to compare the preferred walking, height, weight, and age between the stroke survivors and healthy controls. A mixed analysis of variance (ANOVA) was used to investigate the effect of the different inclines and the effect of stroke in each leg. The differences between the groups and between the incline angles in each dependent variable and its respective variability were evaluated using the F-test. If a significant interaction was found, a *post hoc* Tukey test was used to understand the trends in the results (SPSS 21.0). The effect size is described in the Supplementary Material.

## Results

### Joint Angles

A significant interaction between the effect of incline and the effect of stroke was found in the hip extension (H1) in PL/NL [*F*_(2, 56)_ = 12.82, *p* < 0.001], the hip extension (H1) in NPL/DL [*F*_(2, 56)_ = 7.42, *p* = 0.001], the hip flexion (H2) in PL/NL [*F*_(2, 56)_ = 7.13, *p* = 0.002], the knee extension (K1) in PL/NL [*F*_(2, 56)_ = 3.61, *p* = 0.034], the knee flexion (K2) in PL/NL [*F*_(2, 56)_ = 11.95, *p* < 0.001], and the ankle plantar flexion (A2) in both legs [*F*_(2, 56)_ = 5.68, *p* = 0.006–PL/NL, *F*_(2, 56)_ = 3.99, *p* = 0.0024–NPL/DL, Table 2]. The *post hoc* comparisons between stroke and controls are shown in Table 2. A significant effect of the inclines was found in the hip flexion (H2) in PL/NL [*F*_(2, 56)_ = 57.21, *p* < 0.001] and NPL/DL [*F*_(2, 56)_ = 15.22, *p* < 0.001], the knee extension (K1) of PL/NL [*F*_(2, 56)_ = 4.86, *p* = 0.011], the knee flexion (K2) in NPL/DL [*F*_(2, 56)_ = 6.38, *p* = 0.003], ankle plantar flexion in PL/NL [*F*_(2, 56)_ = 10.51, *p* < 0.0001] and NPL/DL [*F*_(2, 56)_ = 5.68, *p* = 0.006], and ankle dorsiflexion in PL/NL [*F*_(2, 56)_ = 6.44, *p* = 0.003] and NPL/DL [*F*_(2, 56)_ = 3.66, *p* = 0.032].

**Table 2:** Values of the joint angle and joint angle variability in the paretic/non-dominant leg and non-paretic leg/dominant leg in the stroke survivors and controls.

	**0**°****			**3**°****			**6**°****		
	**Stroke (S)**	**Control (C)**	**S vs. C**	**Stroke (S)**	**Control (C)**	**S vs. C**	**Stroke (S)**	**Control (C)**	**S vs. C**
**Paretic Leg/Non-Dominant Leg**
Hip_Extension	−0.83 (7.35)	−5.10 (3.00)	***p** **=*** **0.046**	0.25 (6.76)	−4.41 (3.35)	***p** **=*** **0.023**	1.32 (7.12)	−7.07 (4.14)	***p** **=*** **0.004**
Hip_Flexion	17.92 (7.78)	23.37 (6.15)	***p** **=*** **0.042**	20.57 (8.65)	30.57 (7.21)	***p** **=*** **0.002**	23.18 (9.22)	33.69 (7.96)	***p** **=*** **0.002**
Knee_Extension	−3.88 (4.37)	0.64 (2.66)	***p** **=*** **0.001**	−3.69 (3.84)	2.91 (2.81)	***p** **<*** **0.001**	−3.72 (3.46)	3.05 (3.55)	***p** **<*** **0.001**
Knee_Flexion	42.69 (13.59)	59.42 (4.65)	***p** **<*** **0.001**	42.19 (13.62)	61.75 (4.83)	***p** **<*** **0.001**	40.52 (13.73)	62.80 (4.98)	***p** **<*** **0.001**
Ankle_PlantarFlexion	−7.46 (4.51)	−12.81 (5.15)	***p** **<*** **0.001**	−7.16 (5.19)	−15.13 (7.08)	***p** **=*** **0.001**	−6.76 (5.56)	−17.39 (7.60)	***p** **<*** **0.001**
Ankle_Dorsiflexion	8.13 (5.05)	8.64 (3.21)	NA	10.05 (4.00)	9.53 (3.37)	NA	10.44 (6.42)	9.97 (3.11)	NA
**Non-paretic Leg/Dominant Leg**
Hip_Extension	−2.56 (9.76)	−5.14 (2.99)	NS	−1.79 (8.39)	−4.76 (3.42)	NS	−1.36 (7.26)	−7.28 (4.38)	***p** **=*** **0.011**
Hip_Flexion	25.72 (5.74)	27.26 (8.76)	NA	27.72 (6.49)	32.51 (7.94)	NA	32.36 (6.64)	32.77 (7.46)	NA
Knee_Extension	5.56 (4.01)	1.08 (3.33)	***p** **<*** **0.001**	5.43 (3.79)	3.18 (2.72)	*p =* 0.07	5.88 (3.38)	3.26 (3.72)	*p =* 0.053
Knee_Flexion	52.29 (11.15)	61.23 (3.39)	***p** **<*** **0.001**	53.70 (11.43)	62.63 (3.67)	***p** **<*** **0.001**	54.85 (11.57)	63.32 (5.76)	***p** **<*** **0.001**
Ankle_PlantarFlexion	−5.21 (6.13)	−13.51 (5.28)	***p** **<*** **0.001**	−5.42 (6.68)	−15.51 (6.94)	***p** **=*** **0.001**	−4.94 (8.13)	−18.10 (7.72)	***p** **<*** **0.001**
Ankle_Dorsiflexion	12.37 (7.43)	10.08 (3.93)	NA	13.11 (7.37)	10.83 (3.72)	NA	14.14 (7.46)	11.42 (4.05)	NA
**Paretic/Non-Dominant Leg Variability**
Hip Extension (H1)	2.43 (1.00)	1.55 (0.57)	2.15 (0.82)	1.43 (0.63)	2.25 (1.02)	1.49 (0.65)	NS	***p** **=*** **0.005**	NS
Hip Flexion (H2)	2.70 (1.31)	2.14 (0.94)	2.64 (1.13)	2.05 (0.79)	3.01 (1.68)	2.02 (0.81)	NS	***p*** **=** **0.046**	NS
Knee Extension (K1)	2.61 (1.61)	1.88 (0.48)	2.45 (1.21)	1.67 (0.46)	2.03 (1.12)	1.51 (0.36)	***p** **<*** **0.001**	***p*** **=** **0.05**	NS
Knee Flexion (K2)	3.26 (1.35)	1.89 (0.35)	3.62 (1.93)	1.91 (0.46)	3.69 (2.13)	1.89 (0.53)	NS	***p** **<*** **0.001**	NS
Ankle Plantar Flexion (A2)	2.75 (1.40)	2.71 (1.11)	2.69 (0.93)	2.73 (1.09)	2.30 (1.17)	3.08 (1.16)	NS	NS	NS
Ankle Dorsi Flexion (A1)	1.79 (0.85)	1.54 (0.55)	1.60 (0.74)	1.47 (0.34)	1.68 (0.81)	1.33 (0.34)	NS	NS	NS
**Intact/Dominant Leg Variability**
Hip Extension (H1)	2.53 (1.02)	1.49 (0.36)	2.26 (0.95)	1.56 (0.43)	2.52 (0.89)	1.41 (0.52)	NS	***p** **<*** **0.001**	NS
Hip Flexion (H2)	3.16 (1.77)	2.12 (0.76)	2.71 (0.85)	2.31 (0.92)	3.49 (1.85)	2.34 (0.99)	NS	***p*** **=** **0.017**	NS
Knee Extension (K1)	2.61 (1.19)	2.22 (0.80)	2.37 (1.20)	1.66 (0.49)	2.56 (1.52)	1.57 (0.30)	***p** **=*** **0.048**	***p*** **<** **0.001**	NS
Knee Flexion (K2)	3.98 (1.19)	2.07 (0.55)	3.38 (1.23)	2.07 (0.61)	3.36 (1.03)	1.89 (0.51)	NS	***p** **<*** **0.001**	NS
Ankle Plantar Flexion (A2)	2.80 (1.03)	2.74 (1.04)	3.23 (1.52)	2.84 (1.02)	3.20 (1.82)	2.91 (1.13)	NS	NS	NS
Ankle Dorsi Flexion (A1)	1.87 (0.68)	1,58 (0.47)	1.71 (0.83)	1.53 (0.49)	1.66 (0.77)	1.31 (0.34)	NS	NS	NS

*S, stroke; C, control; NA, no interaction between the effect of stroke and the effect of inclines, thus, post hoc comparisons were not performed; NS, not significant. The meaning of the bold value was that the statistical significance level was reached*.

**Figure 2:**
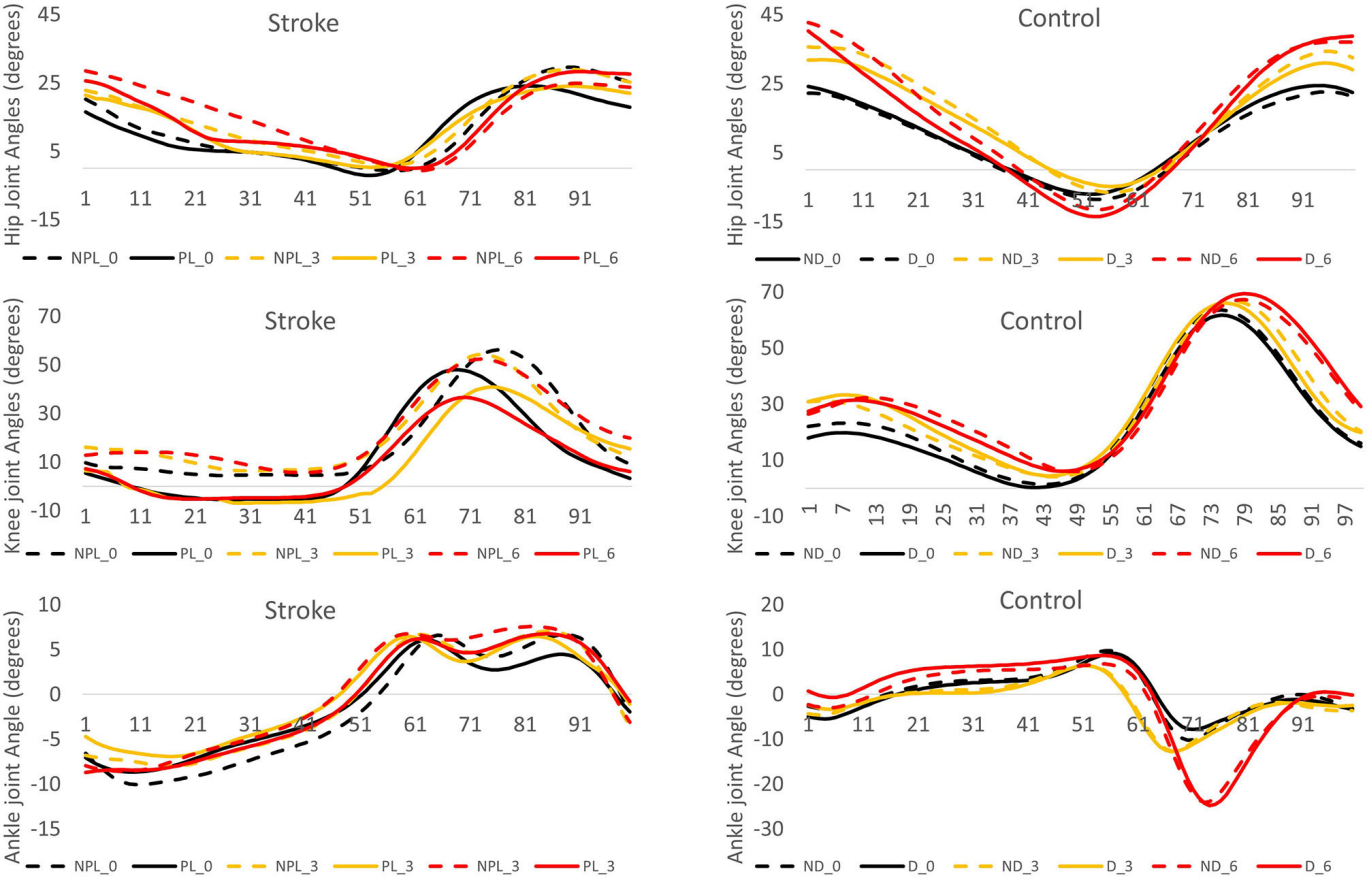
The angle displacement of the hip, knee, and ankle during the gait cycle (expressed in percentage) in the stroke group and control group. The positive values indicate the hip and knee flexion and ankle dorsiflexion. NPL_0: non-paretic leg at 0° incline (black dash line), PL_0: paretic leg at 0° incline (black solid line), NPL_3: non-paretic leg at 3° incline (yellow dash line), PL_3: paretic leg at 3° incline (yellow solid line), NPL_6: non-paretic leg at 6° incline (red dash line), PL_6: paretic leg at 6° incline (red solid line), ND_0: non-dominant leg at 0° incline (black dash line), D_0: dominant leg at 0° incline (black solid line), ND_3: non-dominant leg at 3° incline (yellow dash line), D_3: dominant leg at 3° incline (yellow solid line), ND_6: non-dominant leg at 6° incline (red dash line), D_6: dominant leg at 6° incline (red solid line).

The *post hoc* pairwise comparisons showed that the increasing angles of incline did not significantly increase/decrease the hip extension (H1) in NPL in these patients; however, when walking on the 6° incline, the hip extension (H1) was close to the value of zero in PL. A larger hip flexion in PL/NL was observed when walking on the 3° incline (*p* = 0.003, *p* < 0.001) and 6° incline (*p* < 0.001, *p* < 0.001) compared to when walking on the 0° incline in the patients with stroke and controls, respectively. For knee extension (K1), the increasing inclines did not affect the PL in the stroke survivors. For knee flexion (K2), when walking on the 6° incline, a larger knee flexion was observed in both PL/NPL in the stroke survivors than when walking on the 0° incline (*p* = 0.032, *p* = 0.006, respectively). For the ankle plantar flexion (A2), the increasing angles of the incline did not significantly increase/decrease the ankle plantar flexion in PL/NPL in these patients. Figure 2 shows the graphic representation of the hip, knee, and ankle joint angles during the gait cycle in PL/NL and NPL/DL.

### Joint Angle Variability

No interaction between the effect of stroke and the effect of the inclines was found in any joint angle variability (Table 2). A significant effect of stroke led to a larger hip extension variability in both PL/NL [*F*_(1, 28)_ = 9.28, *p* = 0.005] and NPL/DL [*F*_(1, 28)_ = 15.66, *p* < 0.001], larger hip flexion variability in both PL/NL [*F*_(_1, 28) = 4.35, *p* = 0.046] and NPL/DL [*F*_(1, 28)_ = 6.38, *p* = 0.017], larger knee extension variability in both PL/NL [*F*_(1, 28)_ = 3.974, *p* = 0.05] and NPL/DL [*F*_(1, 28)_ = 5.46, *p* = 0.027], larger knee flexion variability in both PL/NL [*F*_(1, 28)_ = 15.91, *p* < 0.001] and NPL/DL [*F*_(1, 28)_ = 35.71, *p* < 0.001] compared to controls. A significant effect of the inclines was found in the knee extension in both PL/NL [*F*_(2, 56)_ = 10.12, *p* < 0.001] and NPL/DL [*F*_(2, 56)_ = 3.20, *p* = 0.048]. The marginal means indicated that walking on the 6° incline decreased the knee extension variability in both PL/NL (*p* = 0.003) and NPL/DL (*p* = 0.047) compared to walking on the 0° incline.

## Discussion

This study attempted to understand how the joint angles and the respective variabilities changed when walking on different inclines within 80 gait cycles in the stroke survivors and controls. Importantly, the days, since the stroke was limited to 24 months to limit the variances within these patients with stroke (e.g., progression stroke, rehabilitation effect). The results partially had an agreement with our hypotheses that [1] the hip flexion increased as the inclines increased in both the stroke survivors and controls, and [2] higher variabilities in the hip and knee joint angles were observed in the stroke survivors than in the controls. Unexpectedly, the results demonstrated [1] the decrement of the hip extension in the paretic leg, [2] the decrement of the knee flexion in the paretic leg, and [3] no changes in the ankle joint in the stroke survivors in the paretic leg as the inclines increased.

### Alternations in the Peaks of Hip Joint Angle When Walking on Different Inclines

First, the current observations were in line with the previous studies that a decrease in the peaks of hip flexion/extension was observed when walking on the 0° surface compared to controls (30, 31). The previous studies have suggested that ineffectively controlling the hip flexor muscles in pre-swing resulted in a decrease in the hip flexion velocity in early swing and further led to a decrease in the peak of hip flexion (32). Also, a decrease in the peak of hip extension in patients with stroke might be attributed to the insufficient active extensor muscle in the late stance and early swing (33).

In this study, compared to the non-paretic leg, the peaks of hip flexion were 30% at level, 26% at 3° of incline, and 28% at 6° of incline less in the paretic leg in these stroke survivors. Additionally, the peaks of the hip extension were 67% at level, 114% at 3° of incline, and 196% at 6° of incline less in the paretic leg than in the non-paretic leg. Similarly, it has been shown that the hip joint was moderate-to-strongly correlated to the asymmetric gait pattern during walking (34). This asymmetric gait pattern was attributed to the compensatory use of the non-paretic side. Importantly, this asymmetric gait pattern might gradually lead to the potential risk of falling (35). Therefore, to develop treadmill inclination training in stroke survivors, like the previous study (12), a careful consideration of this asymmetric pattern was needed.

Second, walking on different inclines required different body adjustments, resulting in consuming more energy when walking on uphill surfaces than walking on level surfaces (36). Therefore, increasing the peak of hip flexion and decreasing the peak of hip extension as the increasing inclines were the strategies to provide enough power to elevate the lower limb and move the body forward on the treadmill in healthy young adults (37) and in stroke survivors (14). Importantly, the current results found a similar shape of the hip joint angle trajectory compared to the previous studies (12, 14, 37) except the values of these peaks, specifically, the peaks of hip extension (for this study: −0.83 for level, 0.25 for 3° of incline, and 1.31 for 6° of incline compared to the Moreno's study: −12.41 for level, −10.79 for 2.86° of incline, and −9.28 for 5.74° of incline). A rationale could explain these differences: the walking speeds between the studies. In this study, the average walking speed in the stroke survivors was 0.27 m/s compared to 0.71 m/s in Moreno's study. Thus, it is reasonable to speculate that the decrease of the walking speed may reduce the peak of the hip flexion/extension. These speculations have already been confirmed by Kim et al. (38), in whose study, the average speed was 0.33 m/s in the stroke survivors (38).

### Alternations in the Peaks of Knee Joint Angle When Walking on Different Inclines

It is not new that the stroke survivors demonstrated a smaller peak of knee flexion than controls in both legs regardless of the 0°, 3°, or 6° incline. The role of the knee flexion was to ensure that the trajectories of the shank and foot could safely move the body forward on different inclines. Thus, the alternations in the peaks of the knee flexion as increasing the inclines should be minimal (14). Unexpectedly, in this study, the peak of the knee flexion in the paretic leg significantly dropped from 42.69° at the level to 40.52° at the 6° incline in stroke survivors. In contrast, the peak of knee flexion in the non-paretic leg significantly increased from 52.29° at the level to 54.85° at the 6° incline. There might be two rationales for this change. First, we speculated that walking on a higher incline induced a higher asymmetric walking pattern in the stroke survivors. Thus, lowering the peaks of knee flexion might be to avoid overburdening the paretic leg. At the same time, to consistently walk on the 6° incline, the increasing peaks of knee flexion seemingly were inevitable for compensating the paretic leg. Another explanation might be that the different angles of inclines forced the stroke survivors to use different locomotor controls. This speculation was supported by the hypothesis that different distinct controls may exist depending on the different levels of challenges in the same locomotor task (39). In this previous study, two distinct controls were found when taking a single step up a wedge in young adults. These young adults demonstrated one control when the grade of the wedge was below 10° or less and demonstrated another control when the grade of the wedge reached 20° or higher. Thus, in this study, it was reasonable to speculate that several distinct controls might also be performed when the stroke survivors walked on different inclines.

Unexpectedly, a knee hyperextension, genu recurvatum, in the stance phase during the gait cycle in the paretic leg was observed, regardless of the 0°, 3°, or 6° incline. It has been reported that approximately half of the patients with stroke had this symptom. This symptom might be attributed to the ankle plantar flexor weakness (40) and further limited ankle mobility (41). This was the first study to demonstrate that knee hyperextension appeared even when walking on inclines. Perhaps, because the days since stroke was limited, this interesting finding could be observed in this study.

### Alternations in the Peaks of Ankle Joint Angle When Walking on Different Inclines

We did not find a significant effect of incline on the ankle joints in the stroke survivors. This result was confirmed by the previous studies (42, 43) that there were no changes in the gastrocnemius and tibialis anterior muscle activities while walking on different inclines in the stroke survivors. It might be that slow walking reduced in both the dorsi/plantar flexion (44). It was also worth mentioning that in comparison with these previous studies, a slower walking speed could have changed the ankle joint movement trajectories (12, 14, 38).

### Alternations in the Joint Angle Variabilities

As there were no interactions between the effect of incline and the effect of stroke, we could only conclude that a greater variability was found at the hip and the knee in the stroke survivors than healthy controls. These results were mostly in line with the previous studies that the greater joint angle variabilities at the hip, knee, and ankle were observed in patients with neuropathy and patients with multiple sclerosis than controls (18, 19). Based on their suggestions, the slower walking speed was the only factor to lead the greater joint angle variabilities. However, if this hypothesis was true, then a greater variability at all three joint angles should be observed. But, the differences in the joint angle variability at the ankle were not found between the controls and patients in this study. Therefore, we speculated that the walking speed should not be the only factor to increase the joint angle variability, and hemiparetic severity should also be involved. This speculation was suggested by a study of Balasubramanian et al. (45), which indicates that the levels of significant differences in different spatial-temporal gait variabilities depended on the levels of hemiparetic severity.

## Conclusion

The major findings in this study were that [1] an increment of the incline increased the peaks of the hip and knee flexion but decreased the peak of the hip extension and had no impact on the peak of the knee extension in the stroke survivors; [2] the smaller peaks of the hip extension, hip flexion, knee flexion, and ankle plantar flexion in PL/NDL were observed in the stroke survivors than in controls; [3] a greater joint angle variability at the hip and the knee was found in the stroke survivors than in controls.

### Limitations

Please find this section in the Supplementary Material.

## Data Availability Statement

The original contributions presented in the study are included in the article/Supplementary Material, further inquiries can be directed to the corresponding author.

## Ethics Statement

The studies involving human participants were reviewed and approved by Medical Ethics Committee of Shanghai First Rehabilitation Hospital. The Ethics Committee waived the requirement of written informed consent for participation.

## Author Contributions

XZ designed and carried out the experiments. XZ and JC wrote the main text. XZ, YL, CF, ZZ, HL, and GH analyzed the data. TS reviewed and revised the main text. All authors approved the manuscript before submitting it.

## Conflict of Interest

JC is currently employed by JC Movement Science LLC. The remaining authors declare that the research was conducted in the absence of any commercial or financial relationships that could be construed as a potential conflict of interest.

## Publisher's Note

All claims expressed in this article are solely those of the authors and do not necessarily represent those of their affiliated organizations, or those of the publisher, the editors and the reviewers. Any product that may be evaluated in this article, or claim that may be made by its manufacturer, is not guaranteed or endorsed by the publisher.
