# Supplementary material for: The Effect of Inclines on Joint Angles in Stroke Survivors During Treadmill Walking
Source: Front Neurol. 2022 Apr 11;13:850682. doi: 10.3389/fneur.2022.850682 (PMC9037685; doi:10.3389/fneur.2022.850682)
Supplement: Supplementary file 2 [file Table_2.DOCX]

**Effect size**

To investigate the effect size, the partial eta squared method was used. Based on Cohen's guideline, the value of 0.138 is for a large effect size, 0.059 is for a moderate effect size, and is 0.01 for a small effect size.

Partial Eta Squared values were 0.314 for maximum hip extension in paretic/nondominant leg, 0.203 for maximum hip flexion in paretic/nondominant leg, 0.114 for maximum knee extension in paretic/nondominant, 0.125 for maximum ankle plantar flexion. These results indicate the effect size was moderate to large based on the literature (Cohen, 1988).

Reference:

Cohen J. Statistical Power Analysis for the Behavioral Sciences, 2nd ed. Hillsdle. 1988.

**Limitations**

The potential limitation of this study might be the reliability and validity of IMU-based sensors. Specifically, a concern related to the peaks of knee flexion in swing phase was raised (Park and Yoon, 2021). However, if taking a close look on the values, the differences in angles of Knee flexion (IMU: 67.66 ± 5.79 vs. Mocap: 64.58 ± 5.21) and Ankle dorsiflexion and plantar flexion (IMU: 9.63 ± 2.90 vs Mocap: 12.66 ± 2.71; IMU: −23.16 ± 5.09 vs. Mocap: −19.44 ± 3.7) were below 5º. For instrumented motion capture, absolute errors of < 4-5° have been proposed as clinically reasonable (McGinley et al., 2009; Berner et al., 2020B, Seel et al., 2014). Specifically, Berner et al., (2020A) showed that the root-mean-square errors in hip flexion/extension, knee flexion/extension, and ankle dorsi/plantar flexion were within 3º to 4º after adjusting the IMU output by offsetting the calibration trials from optical motion capture system. Same device as the current study (myoMotion, Noraxon) was also used to identify the gait alternation in adults with/without HIV (Berner et al., 2020B). The results suggested that root-mean-square-error and bias were smaller than 5° in the interest points in the current study: Hip flexion – initial contact, Peak hip extension, Knee extension -- stance, knee flexion – stance to swing, ankle plantarflexion and ankle dorsiflexion. Whether these joint angles were adjusted or unadjusted, this previous study did not provide the clear answers. Regardless, this study followed the same procedure for calibration as this previous study, which was suggested manufacturer’s instructions. Importantly, the IMU calibration was performed repeatedly prior to each trial. Therefore, it is reasonable to assume that this device can be used to measure the joint angles in patients with stroke in the current study. Nevertheless, this apparent limitation still needs to be addressed in the future studies by investigating the reliability and validity of this device during gait with large sample size and with different populations.

Another potential limitation was the gait speed in healthy controls and in patients with stroke in the current study. A study used data from 34, 445 older adults aged 65 years or older and indicated that their average speed is 0.92 m/s (Studenski et al., 2011). Additionally, the average walking speed is 0.29 m/s in the acute phase of recovery (McCain et al., 2008). The average walking speeds in both stroke survivors and controls were slower than previous reports. We speculated that 1) these older adults and strokes survivors in China were unfamiliar with treadmill walking, and 2) when they signed the informed consent, they were noticed that the inclined walking trials were given. Therefore, these patients and controls might attempt to use slow walking speed strategies as their preferred walking speed to maintain the balance during inclined walking trial.

Reference:

Berner, K., Cockcroft, J., Morris, L. D., & Louw, Q. (2020A). Concurrent validity and within-session reliability of gait kinematics measured using an inertial motion capture system with repeated calibration. Journal of Bodywork and Movement Therapies, 24(4), 251–260. <https://doi.org/10.1016/j.jbmt.2020.06.008>

Berner, K., Cockcroft, J., & Louw, Q. (2020B). Kinematics and temporospatial parameters during gait from inertial motion capture in adults with and without HIV: A validity and reliability study. BioMedical Engineering OnLine, 19(1), 57. https://doi.org/10.1186/s12938-020-00802-2

McCain, K. J., Pollo, F. E., Baum, B. S., Coleman, S. C., Baker, S., & Smith, P. S. (2008). Locomotor treadmill training with partial body-weight support before overground gait in adults with acute stroke: a pilot study. *Archives of physical medicine and rehabilitation*, *89*(4), 684–691. <https://doi.org/10.1016/j.apmr.2007.09.050>.

McGinley, J. L., Baker, R., Wolfe, R., & Morris, M. E. (2009). The reliability of three-dimensional kinematic gait measurements: a systematic review. *Gait & posture*, *29*(3), 360–369. <https://doi.org/10.1016/j.gaitpost.2008.09.003>.

Park, S., & Yoon, S. (2021). Validity Evaluation of an Inertial Measurement Unit (IMU) in Gait Analysis Using Statistical Parametric Mapping (SPM). *Sensors (Basel, Switzerland)*, 21(11), 3667. <https://doi.org/10.3390/s21113667>.

Seel, T., Raisch, J., & Schauer, T. (2014). IMU-based joint angle measurement for gait analysis. *Sensors (Basel, Switzerland)*, *14*(4), 6891–6909. https://doi.org/10.3390/s140406891

Studenski, S., Perera, S., Patel, K., Rosano, C., Faulkner, K., Inzitari, M., Brach, J., Chandler, J., Cawthon, P., Connor, E. B., Nevitt, M., Visser, M., Kritchevsky, S., Badinelli, S., Harris, T., Newman, A. B., Cauley, J., Ferrucci, L., & Guralnik, J. (2011). Gait speed and survival in older adults. *JAMA*, *305*(1), 50–58. <https://doi.org/10.1001/jama.2010.1923>.
